# A Welcome Chink in Drug Resistance

**DOI:** 10.1371/journal.pbio.1001693

**Published:** 2013-10-29

**Authors:** Robin Meadows

**Affiliations:** Freelance Science Writer, Fairfield, California, United States of America

Given the alarming news about drug-resistant super bugs, it's a relief to know that at least one drug still works even after 50 years of clinical use. Even better, researchers think they finally know why. The drug is amphotericin B and it kills the fungus *Candida albicans*, a common hospital-acquired infection that can be life-threatening. In this issue of *PLOS Biology*, Lindquist and colleagues show that amphotericin B-resistant *Candida* strains have a hard time surviving on their own, let alone against the defenses of a mammalian host.

**Figure:**
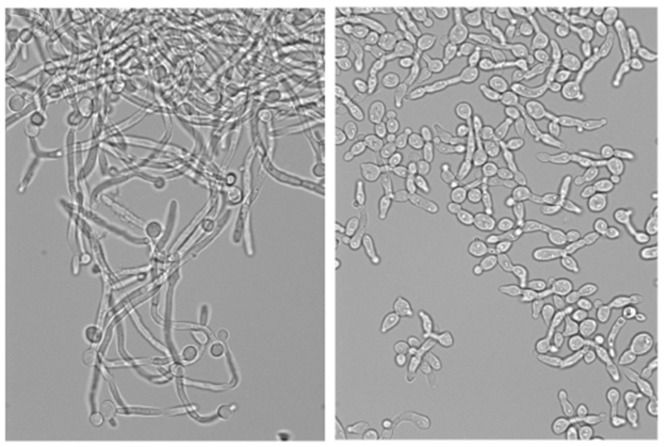
Normal *Candida* (left) forms filaments when stimulated, but amphotericin B-resistant erg2 mutants (right) are “impotent.”

Amphotericin B binds a cholesterol-like component of fungal cell membranes called ergosterol, thereby punching holes in the membrane and killing the cell. The drug is no longer the go-to treatment for *Candida* because it can have nasty side-effects, including fever, tremors, and kidney failure. However, because *Candida* infections often resist modern antifungals, amphotericin B remains a common treatment today.

While extremely rare, a few strains of *Candida* resist amphotericin B. Previous work linked this resistance to mutations in genes (*ERG3* and *ERG11*) for enzymes required to make ergosterol. In the new study, genomic analysis of two resistant strains from patients confirmed the involvement of *ERG3* and *ERG11*, and also added a third gene (*ERG2*) for an enzyme in the ergosterol pathway.

To find more mutations that cause amphotericin B resistance, Lindquist and colleagues used rapid evolution techniques to make a new *Candida* strain that survives this drug. In keeping with the resistant strains from patients, this laboratory-developed resistant strain has a mutation in yet another gene (*ERG6*) involved in making ergosterol.

To solve the mystery of amphotericin B's effectiveness after so many years of clinical use, the researchers investigated Hsp90, a protein that that helps other proteins keep their shapes. Previous experiments have suggested critical roles for Hsp90 in the acquisition of novel traits in species ranging from yeast and plants to fruit flies and humans with tumors. Hsp90 inhibitors block many routes by which pathogenic fungi evolve resistance to triazoles and echinocandins, the current drugs of choice for *Candida*, and likewise overcome resistance in strains that have lost sensitivity to these drugs. The authors found that the same is true for amphotericin B resistance.

But to their surprise, Lindquist and colleagues found that Hsp90 inhibitors also devastate the amphotericin B-resistant strains — even in the absence of treatment with the drug. Because Hsp90 helps stress-response proteins fold properly, the researchers thought stress pathways might be involved. Indeed, while normally activated only during duress, several stress pathways were on all the time in the amphotericin B-resistant strains. These pathways included iron starvation and oxidative stress, both of which are linked to the integrity of fungal membranes.

The next step was to confirm that amphotericin B-resistant *Candida* strains need Hsp90 to stabilize their stress-response proteins. Several fungal stress-response proteins are known to depend on Hsp90 and, as expected, inhibiting these proteins kept the amphotericin B-resistant strains from growing.

After showing that amphotericin B resistance goes hand-in-hand with constant stress response, the researchers asked if this internal stress rendered mutant *Candida* strains helpless to external stresses that come from their host, including fever and attacks to the immune system.. Again, as expected, the amphotericin B-resistant strains hardly grew at elevated temperatures and were hypersensitive to neutrophil attacks.

But that's not all. Amphotericin B resistance also comes with a fungal analog of impotence. While *Candida* normally invades its hosts with long, slender hyphae, the resistant strains hardly made these filaments and could barely penetrate a layer of cultured human skin cells. On top of these findings, mice that were particularly susceptible to *Candida* quickly succumbed to a wild-type strain, yet survived the amphotericin B-resistant strains.

This work offers a solution to the long-standing evolutionary puzzle of why *Candida* has yet to develop resistance to amphotericin B. The results also suggest that new analogs of amphotericin with fewer side effects to patients would have long-lasting therapeutic value. Further, these findings could help anticipate and sidestep the resistance that currently seems inevitable with new antibiotics. For example, we can now design enzyme inhibitors to withstand point mutations, and microbes that become resistant to these designer compounds may, like amphotericin B-resistant *Candida*, find that the cost is too high. As the case of *Candida* and amphotericin B shows, what doesn't kill you won't necessarily make you stronger.

**Vincent BM, Lancaster AK, Scherz-Shouval R, Whitesell L, Lindquist S (2013) Fitness Trade-offs Restrict the Evolution of Resistance to Amphotericin B. doi:10.1371/journal.pbio.1001692** 

